# Supplementary material for: Tlr2 Gene Deletion Delays Retinal Degeneration in Two Genetically Distinct Mouse Models of Retinitis Pigmentosa
Source: Int J Mol Sci. 2021 Jul 22;22(15):7815. doi: 10.3390/ijms22157815 (PMC8435220; doi:10.3390/ijms22157815)
Supplement: Supplementary file 1 [file ijms-22-07815-s001.zip › ijms-1271184-supplementary.pdf]

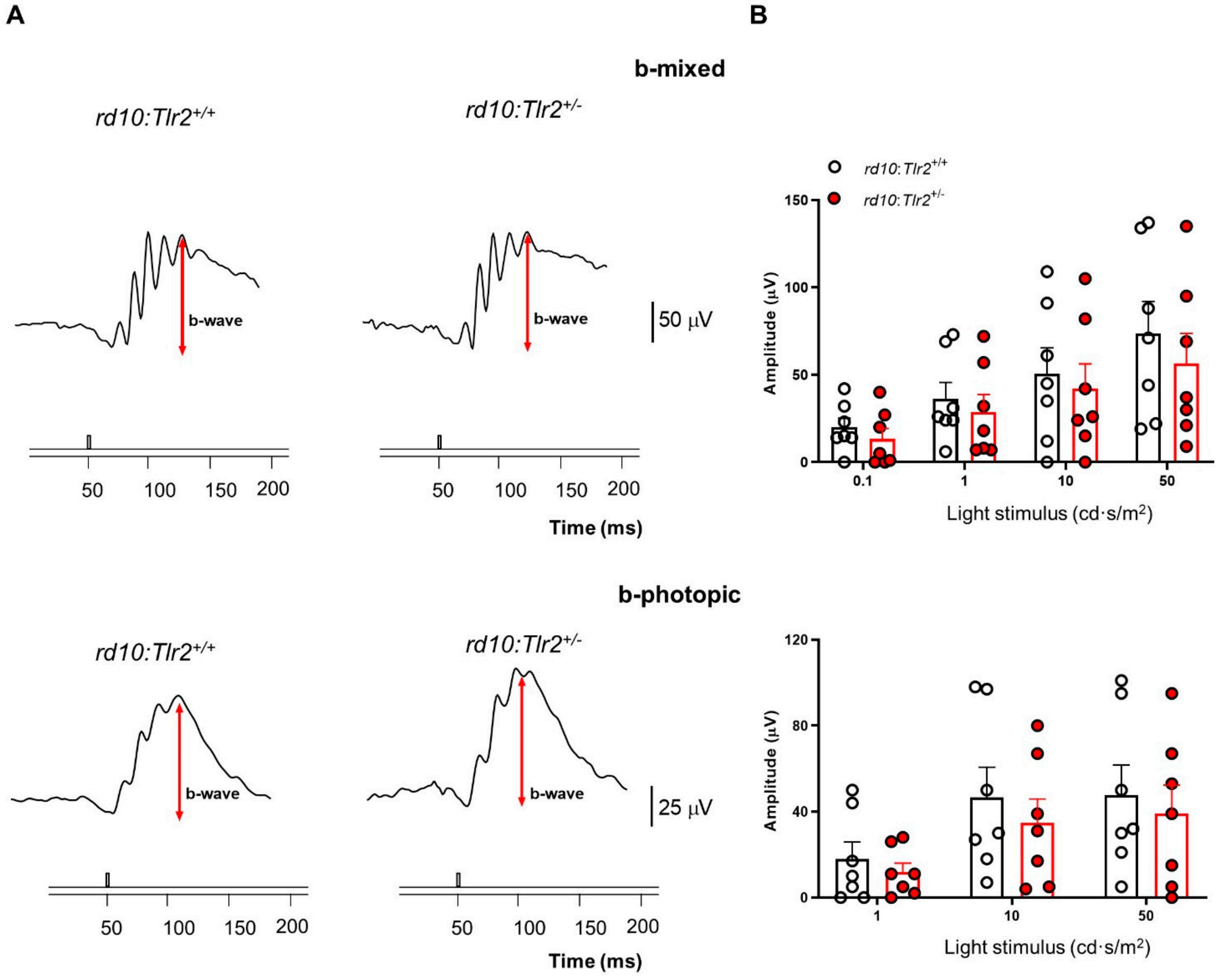

Figure S1. ERG responses of *rd10:Tlr2*$^{+/+}$ and *rd10:Tlr2*$^{+/-}$ mice. (**A**) Representative ERG responses of *rd10:Tlr2*$^{+/+}$ and *rd10:Tlr2*$^{+/-}$ mice (P25) to 50 cd·s/m^2. The value of the scale bar is indicated in the figure. (**B**) Graphs show mean ERG wave amplitudes, plotted as a function of light stimuli. Amplitudes of rod and cone mixed responses (b-mixed waves) to the indicated light intensities were recorded under scotopic conditions after overnight adaptation to darkness. Amplitudes of cone responses (b-photopic waves) to the indicated light intensities were recorded under photopic conditions after 5 minutes of light adaptation (30 cd·s/m^2). Dots represent individual mice and bars represent the mean (+SEM) for each group. n=7 animals per group. Data were analyzed using a 2-way ANOVA.

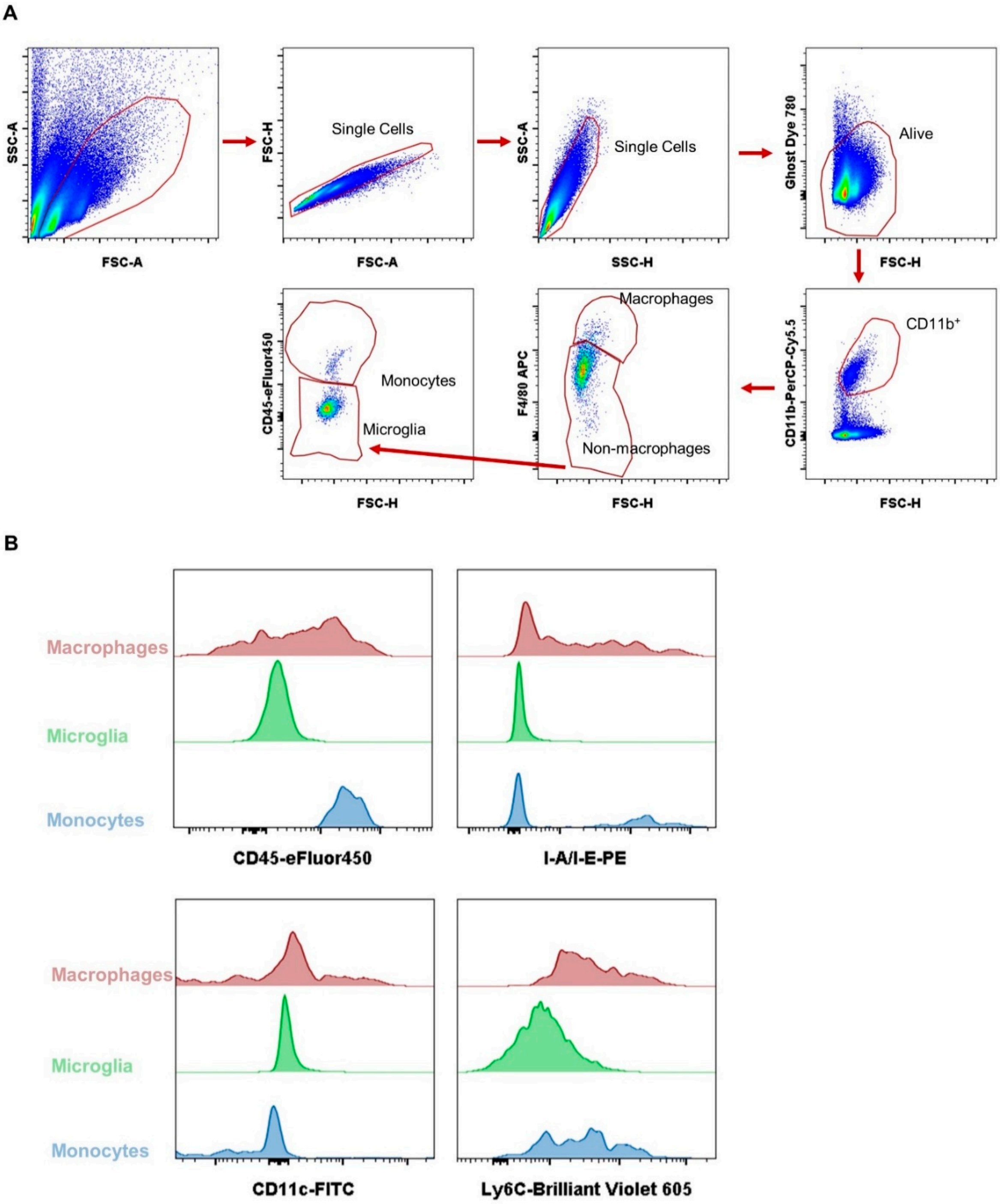

Figure S2. Gating strategy for the identification of myeloid populations. (**A**) Cell doublets and dead cells were excluded from the analysis and CD11b-expressing cells were selected. $CD11b^{+}$ cells expressing high levels of F4/80 were identified as macrophages. The remaining CD11b+ cells were classified according to CD45 expression levels as either microglia ($CD45^{low}$) or monocytes ($CD45^{high}$). (**B**) Analysis of CD45, I-A/I-E (MHC-II), CD11c, and Ly6C expression levels to confirm identity, as described in O'Koren et al [23]. Microglia have a $CD45^{low}$/I-A/I-E^{low}/$CD11c^{low}$ phenotype and lower expression of Ly6C than the other subpopulations. Macrophages have higher levels of CD45, I-A/I-E (MHC-II), CD11c, and Ly6C expression than microglia. Monocytes have the highest levels of CD45 expression, low levels of CD11c expression, and high levels of Ly6C expression.

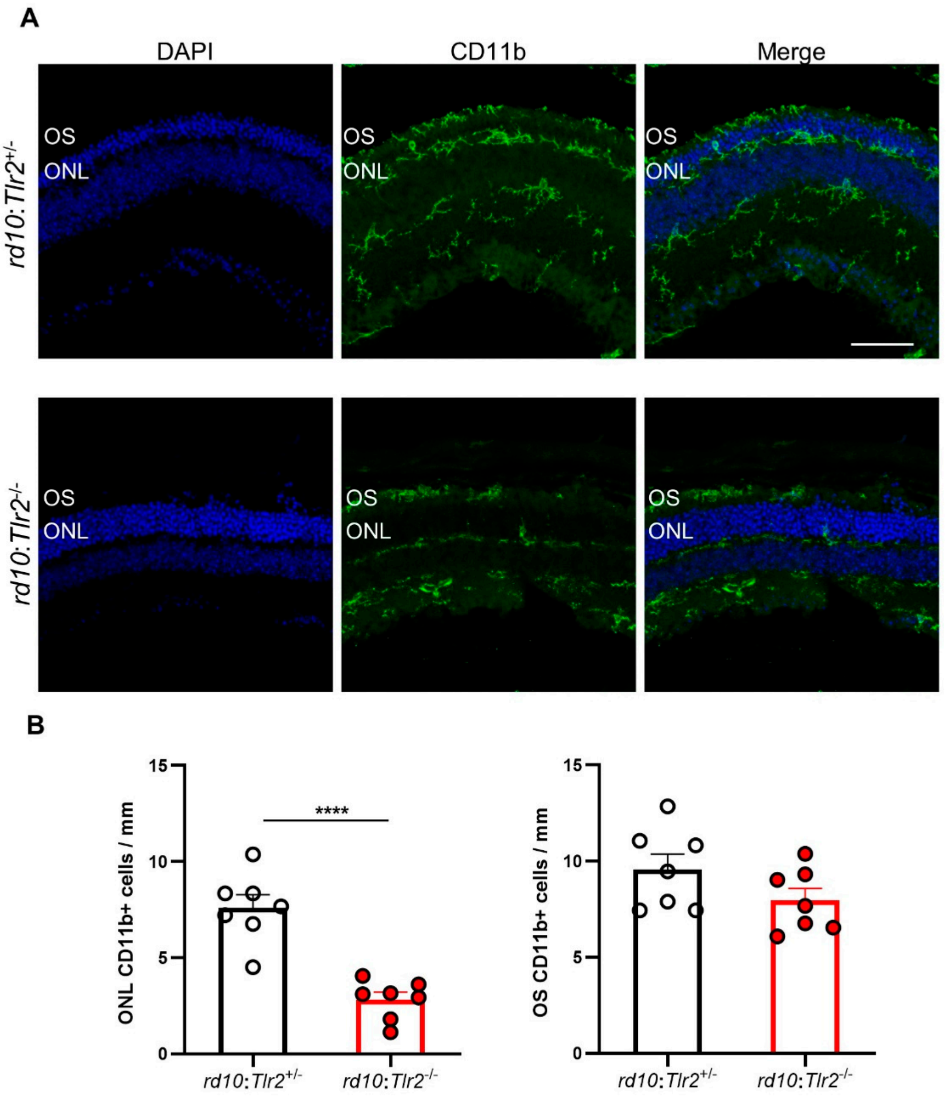

Figure S3. Myeloid cell analysis in *rd10:Tlr2*$^{+/-}$ and *rd10:Tlr2*$^{-/-}$ retinal cryosections. (**A**) Representative confocal optical maximal projections of P36 *rd10:Tlr2*$^{+/-}$ and *rd10:Tlr2*$^{-/-}$ retinal sections. Myeloid cells were stained with CD11b (green) and nuclei were stained with DAPI (blue). Scale bar: 52 μm. **(B)** Quantification of the number of CD11b^{+} cells in the OS and ONL of the retinal sections shown in A. In all cases dots correspond to individual animals and bars represent the mean (+SEM) for each group. OS, outer segment; ONL, outer nuclear layer. n= 7 animals, (3 sections per retina, 6 images per section) **** $p < 0.0001$ (unpaired Student's t test).
